# Novel Fibrillar and Non-Fibrillar Collagens Involved in Fibrotic Scar Formation after Myocardial Infarction

**DOI:** 10.3390/ijms25126625

**Published:** 2024-06-16

**Authors:** María Ortega, Maria Mar Fábrega-García, Tamara Molina-García, Jose Gavara, Elena de Dios, Nerea Pérez-Solé, Víctor Marcos-Garcés, Jaime José Padilla-Esquivel, Ana Diaz, Luis Martinez-Dolz, Manuel Jimenez-Navarro, Cesar Rios-Navarro, Vicente Bodí, Amparo Ruiz-Saurí

**Affiliations:** 1 INCLIVA Biomedical Research Institute, 46100 Valencia, Spain; orcarma@alumni.uv.es (M.O.); tamogar@alumni.uv.es (T.M.-G.); neere_8@hotmail.com (N.P.-S.); vic_mg_cs@hotmail.com (V.M.-G.); amparo.ruiz-sauri@uv.es (A.R.-S.); 2 Department of Pathology, University of Valencia, 46010 Valencia, Spain; marfabregagarcia@gmail.com; 3 Centro de Biomateriales e Ingeniería Tisular, Universidad Politécnica de Valencia, 46022 Valencia, Spain; jose.gavara@outlook.es; 4 Centro de Investigación Biomédica en Red (CIBER)-CV, 28029 Madrid, Spain; elenaddll@gmail.com (E.d.D.); luis.v.martinez@uv.es (L.M.-D.); mjimeneznavarro@uma.es (M.J.-N.); 5 Cardiology Department, Hospital Clínico Universitario, 46010 Valencia, Spain; 6 Anatomic Pathology Department, Hospital Clínico Universitario, 46010 Valencia, Spain; jpadillaesquivel@gmail.com; 7 Unidad Central de Investigación Médica, University of Valencia, 46010 Valencia, Spain; ana.diaz@uv.es; 8 Cardiology Departament, Hospital Universitario Politécnico La Fe, 46026 Valencia, Spain; 9 Instituto de Investigación Sanitaria La Fe, 46026 Valencia, Spain; 10 Servicio de Cardiología y Cirugía Cardiovascular-Área del Corazón, Hospital Universitario Virgen de la Victoria, 29010 Málaga, Spain; 11 Instituto de Investigación Biomédica de Málaga y Plataforma en Nanomedicina (IBIMA Plataforma BIONAND), 29590 Málaga, Spain; 12 Departamento de Medicina y Dermatología, Facultad de Medicina, Universidad de Málaga, 29010 Málaga, Spain; 13 Department of Medicine, University of Valencia, 46010 Valencia, Spain

**Keywords:** myocardial infarction, fibrosis, collagen

## Abstract

Following myocardial infarction (MI), adverse remodeling depends on the proper formation of fibrotic scars, composed of type I and III collagen. Our objective was to pinpoint the participation of previously unreported collagens in post-infarction cardiac fibrosis. Gene (qRT-PCR) and protein (immunohistochemistry followed by morphometric analysis) expression of fibrillar (types II and XI) and non-fibrillar (types VIII and XII) collagens were determined in RNA-sequencing data from 92 mice undergoing myocardial ischemia; mice submitted to permanent (non-reperfused MI, n = 8) or transient (reperfused MI, n = 8) coronary occlusion; and eight autopsies from chronic MI patients. In the RNA-sequencing analysis of mice undergoing myocardial ischemia, increased transcriptomic expression of collagen types II, VIII, XI, and XII was reported within the first week, a tendency that persisted 21 days afterwards. In reperfused and non-reperfused experimental MI models, their gene expression was heightened 21 days post-MI induction and positively correlated with infarct size. In chronic MI patients, immunohistochemistry analysis demonstrated their presence in fibrotic scars. Functional analysis indicated that these subunits probably confer tensile strength and ensure the cohesion of interstitial components. Our data reveal that novel collagens are present in the infarcted myocardium. These data could lay the groundwork for unraveling post-MI fibrotic scar composition, which could ultimately influence patient survivorship.

## 1. Introduction

Myocardial infarction (MI) consists of the acute thrombotic occlusion of a coronary artery, leading to an abrupt reduction in oxygen and nutrient supply to the downstream myocardium. Although the timely reopening of the culprit vessel (ideally by percutaneous intervention) is mandatory to limit infarct size, substantial changes in the infarcted myocardium nonetheless occur [1,2,3]. During post-MI cardiac healing, a sterile and well-orchestrated inflammatory response is rapidly initiated to remove necrotic cell debris from the MI zone. Afterwards, the deposition of myofibroblast-derived collagen fibers results in solid fibrotic scar formation [1,3,4]. This process needs to be precisely controlled in terms of timing and position to avoid excessive left ventricular dilatation.

Experimental and clinical studies indicate that type I and III collagens are the more abundant fibers participating in the post-MI fibrotic scar composition [5,6]. In fact, not only their presence but also their spatial distribution is essential to minimize the appearance of arrhythmias [7]. A recent review observed the involvement of up to eight different collagens (including types I, III, IV, V, VI, XII, XIV, and XVIII) in the fibrotic scar of human samples or experimental models of myocardial ischemia [8].

However, the emergence of next-generation sequencing approaches has revolutionized biomedical research by providing abundant omics data for understanding the molecular mechanisms underlying diseases. In this specific setting, a recent meta-analysis from our group using RNA-sequencing data from 92 mice undergoing permanent occlusion of the coronary artery suggested that up to 26 collagen subunits were probably implicated in the MI context [9]. Given that collagen composition within the fibrotic scar actively influences MI patient prognosis and left ventricular dilation, exploring novel subunits implicated in post-MI pathophysiology is of great interest.

Using previously published data at the gene level as a springboard, our aim was to gain further insight into the composition of post-MI fibrotic scars by assessing the existence of previously unreported collagens, specifically types II, VIII, XI, and XII, in three different scenarios: (i) meta-analysis of transcriptomic data from 92 mice undergoing different times of coronary artery occlusion; (ii) experimental mouse models of reperfused and non-reperfused MI; and (iii) myocardial samples isolated from chronic MI patients.

## 2. Results

### 2.1. Transcriptomic Expression of New Collagen Subunits Based on RNA-Sequencing Datasets from Mice Submitted to Non-Reperfused MI

First, the transcriptomic expression of these newly described collagen subunits was evaluated in RNA-sequencing datasets from mice undergoing different ischemia times. Using the search term “myocardial infarction”, a total of 15,337 investigations were found for this purpose; only 143 datasets were deemed eligible. After removing duplicate series (n = 51), 84 were excluded for the following reasons: knock-out mice (n = 24), single-cell or single-nuclear RNA-sequencing research (n = 28), evaluation of non-infarcted area (n = 11), mice submitted to pharmacological treatment (n = 14), newborn mice (n = 3), insufficient dataset information (n = 2), and mice submitted to 4 days of ischemia (n = 2).

Finally, eight datasets (including 92 animals) were employed in the meta-analysis. Animals were divided into control mice (n = 30, without MI induction) and 62 submitted to different times of coronary occlusion: 6 h (n = 8), 1 day (n = 16), 3 days (n = 10), 7 days (n = 7), 14 days (n = 10), and 21 days (n = 11). The datasets, the associated publication, and the number of samples included in each study group are compiled in Table 1 [10,11,12,13,14,15].

**Figure 1:**
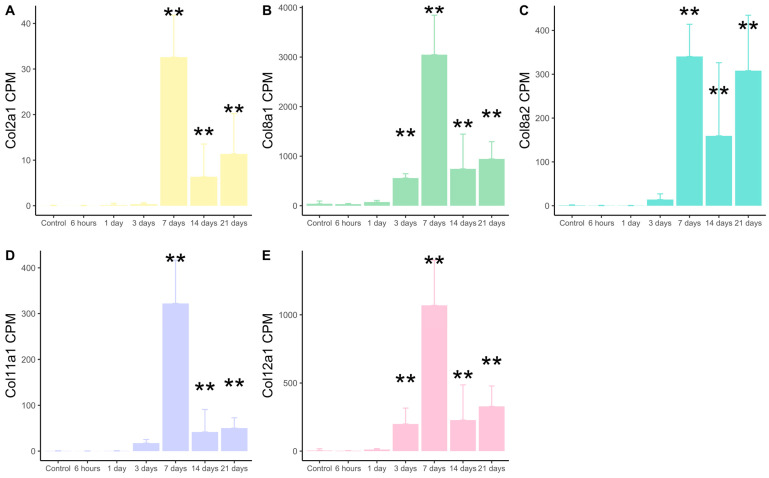
Transcriptomic expression of types II, VIIIa1, VIIIa2, XI, and XII subunits at different time points after ischemia onset. RNA-sequencing datasets were analyzed from 62 animals submitted to 6 h (n = 8), 1 day (n = 16), 3 days (n = 10), 7 days (n = 7), 14 days (n = 10), and 21 days (n = 11) of myocardial ischemia, and 30 controls (without myocardial infarction induction). CPM of type II (**A**), VIIIa1 (**B**), VIIIa2 (**C**), XI (**D**), and XII (**E**) collagen subunits are expressed. ** *p*-value < 0.01 vs. control. CPM: counts per million.

We next sought to pinpoint dynamic alterations in the expression of type II, VIIIa, VIIIb, XI, and XII collagen subunits detected in the infarcted myocardium. In comparison to controls, increased transcriptomic expression of type VIIIa and XII collagen subunits was observed 3 days after ischemia onset, while type II, VIIIb, and XI subunits were overrepresented from day 7 post-MI onwards (Figure 1).

Indeed, transcriptomic changes in important genes encoding type I and III collagens and key regulators of the fibrotic response (e.g., *ccn2*, *acta2*, and *tgfb1*) were also scrutinized (Table 2). According to our results, the transcriptomic levels of *ccn2* and *tgfb1* were heightened in the first hours after the onset of coronary ischemia. As for collagen subunits, the transcriptomic expression of *col3a1* and *col1a1* levels were augmented from day 3 onwards (Table 2). Taking all these results together, the transcriptomic expression of these novel subunits is upregulated within the first week after ischemia onset until chronic phases (21 days).

### 2.2. Involvement of New Collagen Subunits in Non-Reperfused and Reperfused Models of MI

To confirm these results, the mRNA expression of type II, VIIIa1, VIIIa2, XI, and XII collagens were quantified in murine hearts isolated 21 days after MI induction. Animals submitted to permanent coronary ligation (non-reperfused MI) and transient 45 min coronary occlusion followed by reperfusion (reperfused MI) were used.

First, the mRNA levels of genes encoding type I and III collagens and key regulators of the fibrotic response (e.g., *ccn2*, *acta2*, and *tgfb1*) were calculated in both experimental models of MI (reperfused and non-reperfused) and control animals to confirm the presence of myocardial fibrosis. The mRNA levels of *col1a1*, *col3a1*, *tgfb1*, and *acta2* were heightened in both mouse models of MI (Table 3) and displayed a direct correlation with the magnitude of infarct size evaluated by Masson’s Trichrome staining (Table 4).

**Figure 2:**
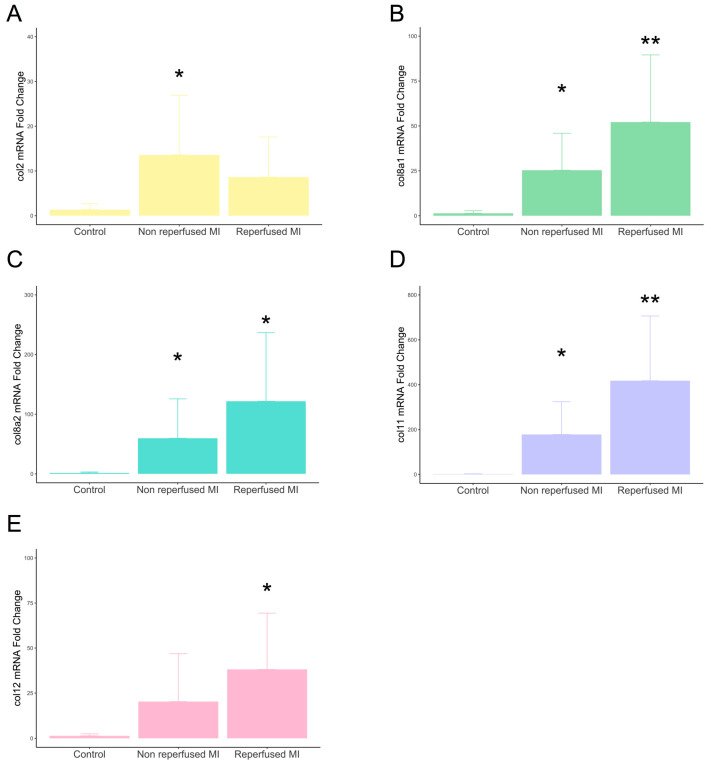
mRNA expression of type II, VIIIa1, VIIIa2, XI, and XII subunits in experimental controlled murine models of MI. Mice submitted to permanent coronary ligation (non-reperfused MI, n = 8) and transient 45 min coronary occlusion followed by reperfusion (reperfused MI, n = 8) and a control group (n = 6) were used. Transcriptomic levels of type II (**A**), VIIIa1 (**B**), VIIIa2 (**C**), XI (**D**), and XII (**E**) collagen subunits at day 21 after MI induction. Continuous normally distributed data are expressed as mean ± SD and were analyzed by unpaired Student’s *t*-test. * *p*-value < 0.05 ** *p*-value < 0.01 vs. control. MI: myocardial infarction.

Gene expression of type VIIIa1, VIIIa2, and XI subunits displayed enhanced mRNA expression in the infarcted myocardium derived from both non-reperfused and reperfused models compared to controls. The transcriptomic levels of type II subunits were only augmented in the non-reperfused MI group, while type XII was overrepresented in reperfused, but not in non-reperfused, animals (Figure 2).

**Figure 3:**
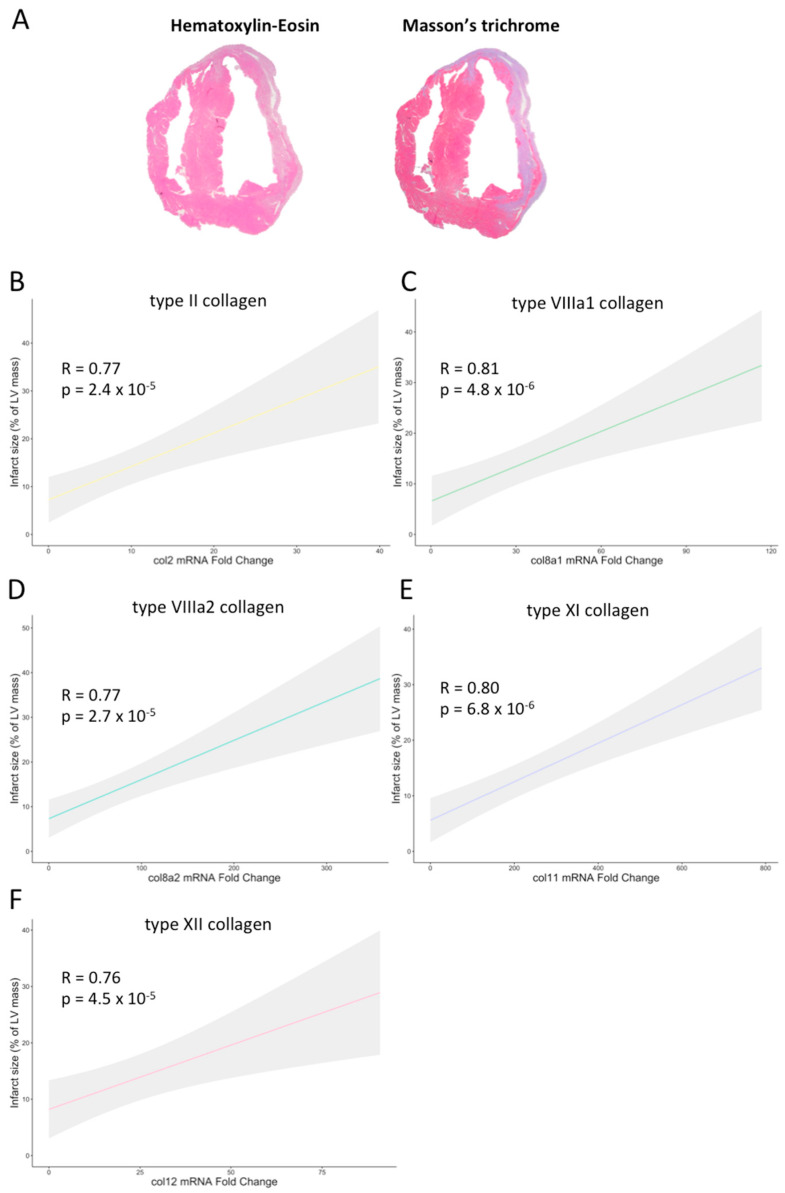
Association of infarct size with the mRNA expression of type II, VIIIa1, VIIIa2, XI, and XII collagen subunits in experimental controlled murine models of myocardial infarction. (**A**) Representative pictures of hematoxylin–eosin (left panel) and Masson’s Trichrome (right panel) staining performed in a heart submitted to permanent coronary ischemia. In mice submitted to permanent (non-reperfused) or transient (reperfused) coronary ischemia, the extension of infarct size showed a positive association with the transcriptomic levels of type II (**B**), VIIIa1 (**C**), VIIIa2 (**D**), XI (**E**), and XII (**F**) collagen subunits. LV: left ventricle.

A direct association was detected between the gene expression of these five collagen subunits and histological-derived infarct size. The Spearman rank-order correlations are as follows: types II (0.77; *p*-value < 0.001), VIIIa1 (0.81; *p*-value < 0.001), VIIIa2 (0.77; *p*-value < 0.001), XI (0.80; *p*-value < 0.001), and XII (0.76; *p*-value > 0.001) (Figure 3). Collectively, these data demonstrated the implication of these new subunits at the gene level in experimental models of MI, in which mRNA levels were positively correlated with fibrotic scar extension.

### 2.3. Participation of Novel Collagen Subunits in Fibrotic Scar from Patients with Chronic MI

Once the involvement of these new collagen subunits was confirmed in experimental models of MI, we next corroborated these results in human patients. This study examined cardiac samples taken from eight patients who had been diagnosed with chronic MI (more than six months). Table 5 lists their initial features as well as the results of their autopsy. Briefly, the mean age of the eight patients, six of whom were male, was 74 ± 7 years, and all samples showed evidence of fibrosis.

**Figure 4:**
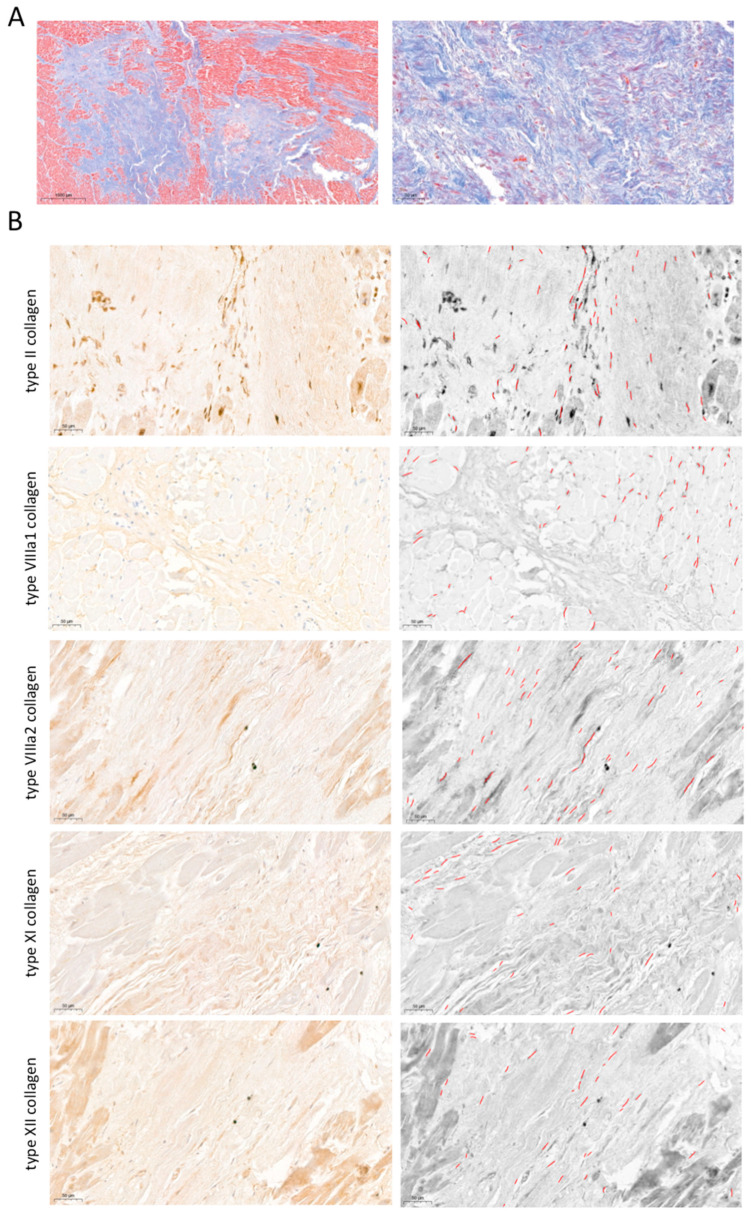
Presence of type II, VIIIa1, VIIIa2, XI, and XII subunits in fibrotic scar of patients with chronic myocardial infarction. (**A**) Representative pictures of Masson’s Trichrome stain at macroscopic (left panel) and 40× (right panel) magnification from infarcted samples isolated from patients at the chronic (more than 6 months) stage following MI. (**B**) Representative images from human infarcted tissue (left panels) stained with specific markers against type II (upper), VIIIa1 (upper-middle), VIIIa2 (middle), XI (lower-middle), and XII (lower) subunits and their corresponding morphometric analysis using the software Image ProPlus 7.0 software (Media Cybernetics Inc., Rockville, MD, USA) (right panels). Bars indicate 50 μm.

The presence of type II, VIIIa1, VIIIa2, XI, and XII subunits at the protein level was determined in a total of 200 different areas. Data were expressed as the number of fibers and the percentage per field occupied by positive elements (Figure 4). Immunohistochemistry analysis revealed the presence of type II (21.5 ± 7.8 fibers occupying 0.26 ± 0.11% of the field), VIIIa1 (36.9 ± 19.4 fibers occupying 0.51 ± 0.53% of the field), VIIIa2 (29.8 ± 12.4 fibers occupying 0.33 ± 0.11% of the field), XI (14.7 ± 3.7 fibers occupying 0.15 ± 0.04% of the field), and XII (21.1 ± 8.2 fibers occupying 0.20 ± 0.07% of the field) collagen subunits in the post-MI fibrotic myocardium.

Next, the mRNA levels of these five collagen subunits were evaluated using qRT-PCR, with similar findings to the control myocardium isolated from patients, with no evidence of cardiac fibrosis at either macroscopic or microscopic levels. These results indicated that although the mRNA expression of these newly reported collagen subunits was already upregulated a few hours after ischemia onset, their gene expression was unaltered at the chronic stage (more than 6 months) after MI.

### 2.4. Gene Ontology Enrichment Analysis to Predict the Function of the Novel Subunits in the Context of MI

**Figure 5:**
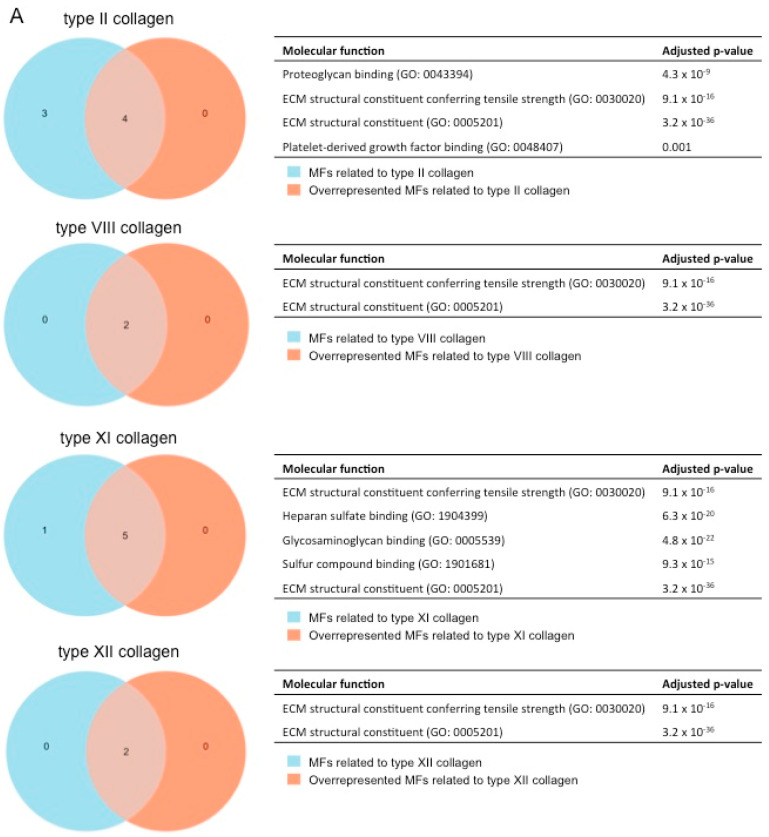

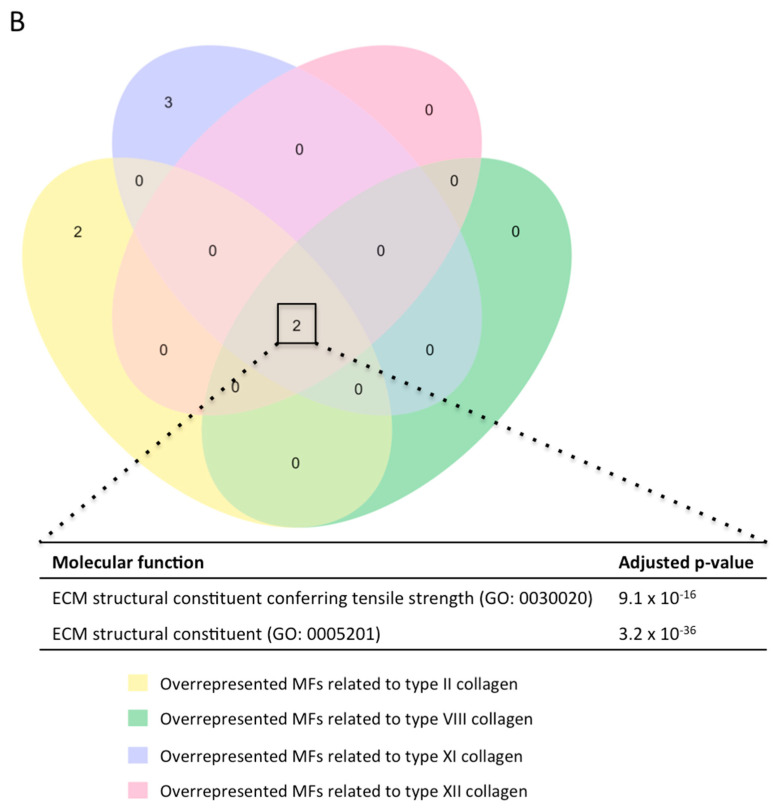
(**A**) GO enrichment analysis to predict the MF of type II, VIII, XI, and XII collagen in the context of myocardial infarction. The diagrams represent the MF terms of the GO analysis related to type II (upper), VIII (upper-middle), XI (lower-middle), and XII (lower) collagens (blue areas) that are overrepresented in our meta-analysis (orange areas) together with the adjusted *p*-value. (**B**) GO enrichment analysis to predict the MF of type II, VIII, XI, and XII collagen in the pathophysiology of myocardial infarction. The Venn diagram shows the MF terms of the GO analysis, which are upregulated in the four evaluated collagens. ECM: extracellular matrix. GO: Gene Ontology. MF: molecular function.

After showing the involvement of these novel collagen subunits in MI, we next performed a functional enrichment analysis with the molecular function (MF) terms of Gene Ontology (GO) analysis to hypothesize their function. Briefly, we identified which MFs related to each collagen subunit were overrepresented in our meta-analysis (with an adjusted *p*-value lower than 0.01) (Figure 5).

For the type II (fibril-forming collagen) subunit, the following four MFs were overrepresented: proteoglycan binding (GO:0043394), extracellular matrix constituent conferring tensile strength (GO:0030020), extracellular matrix constituent (GO: 0005201), and platelet-derived growth factor binding (GO:0048407) (Figure 5A).

When evaluating MFs related to the fibrillar type XI collagen, a total of five different MFs were detected: extracellular matrix constituent conferring tensile strength (GO:0030020), heparan sulfate binding (GO:1904399), glycosaminoglycan binding (GO:0005539), sulfur compound binding (GO:1901681), and extracellular matrix constituent (GO:0005201).

In terms of non-fibrillar collagen (types VIII and XII), the MFs extracellular matrix constituent conferring tensile strength (GO:0030020) and extracellular matrix constituent (GO:0005201) were upregulated in our meta-analysis (Figure 5A), and these MFs were likewise related to all the new collagen subunits evaluated in our study (Figure 5B).

Overall, four new collagen subunits (types II, VIII, XI, and XIII) participate in the pathophysiology of MI by providing tensile strength to the infarcted tissue (Figure 5B), and fibril-forming collagens also ensure the cohesion of different extracellular matrix (ECM) components (i.e., heparan sulfate, glycosaminoglycans, and proteoglycans).

## 3. Discussion

### 3.1. The Role of the Fibrotic Process in MI Pathophysiology

MI consists of acute thrombotic occlusion of a coronary artery, provoking a sudden reduction in nutrient and oxygen supply to the downstream myocardium [16]. Consequently, an inflammatory response is rapidly initiated to remove necrotic cells and damaged ECM components and activate intracellular pathways for quick tissue repair [4,8]. The post-MI wound-healing process consists of a series of events involving complex interactions between various cell types, including myofibroblasts and endothelial cells, leading to the formation of a solid fibrotic scar mainly composed of collagen fibers [6,8].

This process must be perfectly regulated, given that post-MI fibrosis has been reported to exert a dual effect on cardiac structure. While collagen deposition is undoubtedly essential to prevent ventricular dilation and compromised systolic function, massive collagen accumulation nonetheless provokes tissue stiffness, lack of myocardial elasticity, and heightened incidence of arrhythmias. In fact, approximately 30% of MI patients develop adverse cardiac remodeling due to massive collagen accumulation, which correlates with heart failure occurrence [17,18,19]. Consequently, it is crucial to have a more comprehensive knowledge of the players participating in post-MI fibrosis for heightening patient survival rates.

### 3.2. Collagen Fibers Participating in Fibrotic Scar Formation following MI

Collagen proteins, active players in post-MI cardiac fibrosis, can be divided into two broad categories. Fibrillar collagens provide mechanical strength and three-dimensional frameworks for cardiac organization [20]. The main fibril-forming collagens are type I and III subunits, which are considered the principal components of the cardiac ECM at the post-infarction fibrotic stage [21]. In fact, its spatial orientation is thought to be essential for proper left ventricular function [7]. Non-fibrillar collagens, meanwhile, play a regulatory role in anchoring and organizing the ECM meshwork via association with type I and III subunits [22]. Specifically, type IV, VI, XIV, and XVIII subunits have already been described to be involved in experimental models of MI and ischemic heart failure [8].

Our group recently conducted a meta-analysis utilizing RNA-sequencing data derived from mice undergoing myocardial ischemia. The results revealed the overrepresentation of 42 genes that encode 26 different collagen subunits within the post-MI fibrotic scar [9]. Of these, up to four collagens, including fibrillar (II and XI) and non-fibrillar types (VIII and XII), are understudied in the context of MI. Therefore, our investigation aims to pinpoint the presence of these four novel collagen subunits in three different scenarios: (i) meta-analysis of transcriptomic data from 92 mice undergoing different times of coronary artery occlusion; (ii) mice undergoing permanent coronary ligation or transient coronary occlusion; (iii) autopsies from chronic MI patients.

### 3.3. Unraveling the Participation of Novel Collagen Subunits in the MI Scenario

Firstly, the mRNA expression of these subunits at the initial stages of ischemia was evaluated in RNA-sequencing transcriptomic datasets from mice submitted to different times of coronary blockade. According to our results, an upregulation in the gene levels of type II, VIII, and XII subunits is detected in the first week after ischemic insult. However, the transcriptomic levels of all new collagen subunits are augmented from day 3 onwards, peaking 7 days after MI induction. To corroborate these data, we evaluated the mRNA of these previously unreported collagen subunits in infarcted tissue at the subacute stage (21 days) derived from two controlled mouse models of MI (reperfused and non-reperfused). Our data show that the transcriptomic levels of types VIII and XI are heightened in both models. Contrarily, gene levels of type II subunits are augmented in the non-reperfused MI group, while type XII is only upregulated in the reperfused group.

**Figure 6:**
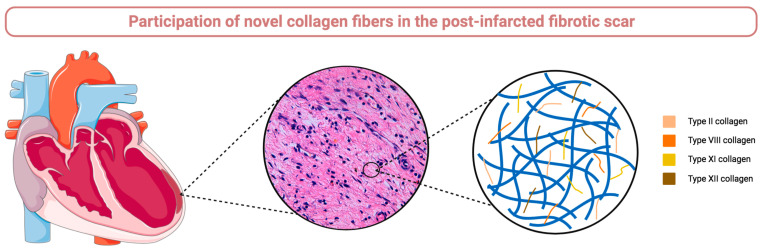
Graphical abstract. The participation of type II, VIII, XI, and XII collagen subunits is implicated in fibrotic scar formation after myocardial infarction as reflected by their mRNA upregulation in infarcted myocardium isolated from experimental models and their protein presence in autopsies isolated from patients diagnosed with chronic infarction.

Following on from this, our next goal was to characterize these subunits in human samples isolated from patients with chronic MI. Protein presence in the infarcted myocardium was studied using immunohistochemistry staining followed by morphometric analysis. Based on our data, the involvement of type II, VIII, XI, and XII collagen subunits is detectable in human autopsies, with a greater presence of non-fibrillar collagens than fibril-forming collagens (types II and XI). A proteomics analysis in infarcted myocardium isolated from swine undergoing 120 min of ischemia followed by 15 or 60 days of reperfusion revealed the overrepresentation of type XII, but not type XI, collagen subunits in both MI groups compared to controls. Contrarily, no data were reported regarding the presence of type II or VIII subunits [23]. Our data also showed that the gene expression of these four subunits was unaltered in human infarcted myocardium compared to control hearts. Overall, although these new collagen subunits participate in the infarcted myocardium at the protein level, their transcriptomic levels remain unaltered in an already-established scar. Collectively, these novel collagen subunits participate in the fibrotic scar at chronic phases following MI (Figure 6), but their mRNA expression is already upregulated within a few hours after ischemia onset.

In a recent review, Frangogiannis and colleagues reported the involvement of up to eight different collagens (including types I, III, IV, V, VI, XII, XIV, and XVIII) in ischemic heart failure [8]. However, although our results provide insight into new players in fibrotic scar formation following infarction, further clinical and translational research is necessary for a thorough comprehension of the impact of these new elements in the pathophysiology of post-MI fibrosis.

### 3.4. The Role of New Collagen Subunits in the Post-Infarction Fibrosis

According to the Human Protein Atlas, fibroblasts are reported to display an enlarged mRNA expression of col2a1, col8a1, col8a2, and col12a1, whereas there are no data on cardiac resident cells capable of secreting col11a1. However, studies reporting their implication in MI pathophysiology are scarce.

In terms of fibrillar collagens, type II collagen is the main component of cartilage. Despite its transient expression at cardiac valve morphogenesis, to our knowledge, no expression has been demonstrated in human adult hearts. In cardiovascular diseases, a more elevated presence of type II collagen is suggested around the atherosclerosis-derived calcium deposit. The type XI subunit, a fibril-forming collagen, regulates the fibrillogenesis of type I and type II collagens and is expressed in locations such as articular cartilage, tendons, and trabecular bone. In the cardiac tissue, type XI collagen takes part in developing heart valves and left ventricular trabeculae, and its involvement in pathological situations has been studied in patients with pancreatic ductal adenocarcinoma and biliary tract cancer. Specifically in the MI scenario, after performing GO enrichment analysis, types II and XI probably participate in conferring tensile strength to the cardiac interstitium as well as binding different ECM elements including heparan sulfate, glycosaminoglycans, platelet-derived growth factor, and sulfur compounds.

Regarding non-fibrillar collagens, the type VIII subunit (included in the subfamily of network-forming collagens) participates in cardiogenesis, while reduced expression is reported in physiological adult myocardium. In cardiovascular diseases, type VIII collagen has been implicated in cardiac dilatation-derived heart failure following pressure overload [24], carotid artery distensibility [25], and atrial fibrillation [26]. In fact, fibroblasts isolated from col8-knock-out mice displayed lower expression of transforming growth factor-β and α-smooth muscle actin, key players in post-MI cardiac fibrosis [24]. Type XII is classified as fibril-associated collagen with interrupted triple helices, forms tendons, and is overrepresented in human hearts as well as in the epicardium of zebrafish [27]. Its participation has also been shown in several pathological conditions, including idiopathic interstitial pneumonia and pleomycin-induced pulmonary fibrosis in mice [28]. In the specific case of cardiovascular diseases, type XII collagen is upregulated in resident cardiac progenitor cells from transgenic heart failure mice and zebrafish hearts submitted to cryoinjury [27], but its role following MI is understudied. According to our analysis, both non-fibrillar collagens (types VIII and XII) may participate in conferring tensile strength to the cardiac ECM. However, further bench and bedside research is necessary for an in-depth elucidation of their role in MI pathophysiology.

## 4. Materials and Methods

### 4.1. Meta-Analysis of RNA-Sequencing Data

A dataset search of studies up to September 2021 using the term “myocardial infarction” was carried out in the Gene Expression Omnibus (GEO) database [29] to select studies for meta-analysis.

Studies conducted in Mus musculus, animals undergoing non-reperfused MI (permanent coronary artery ligation) or sham, and investigations utilizing bulk RNA-sequencing analysis from the infarcted myocardium were the inclusion criteria. Exclusion criteria included single-cell or single-nucleus RNA-sequencing research, studies involving newborn mice or exploring pharmaceutical or mechanical interventions, analyses conducted in blood or non-infarcted myocardium, and investigations that were not properly filtered by GEO filters.

**Figure 7:**
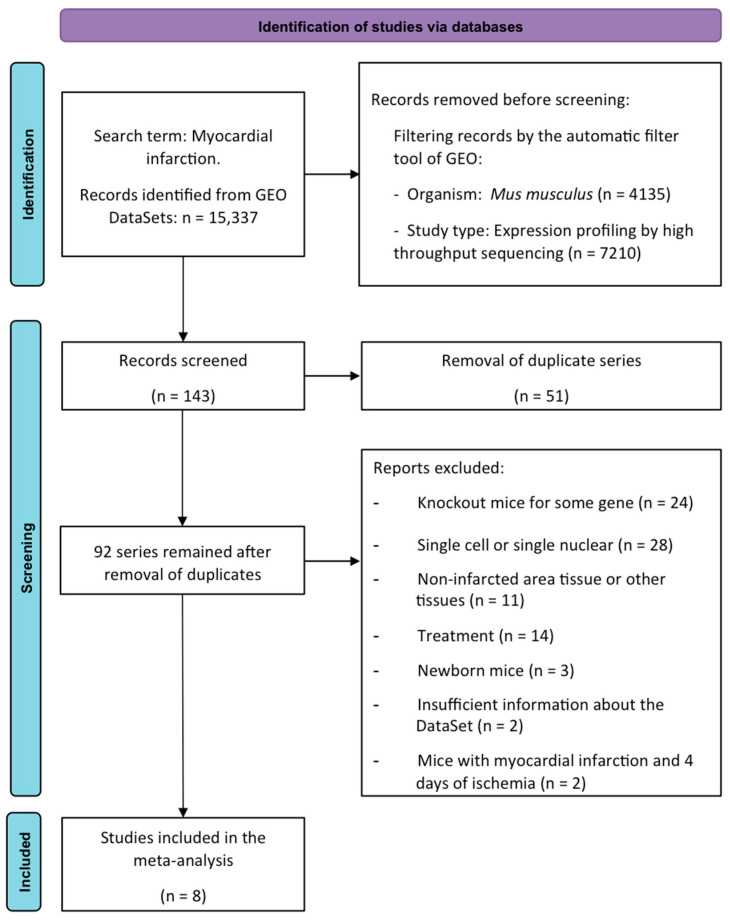
PRISMA meta-analysis flow diagram. GEO: Gene Expression Omnibus.

The PRISMA Flow Diagram (Figure 7) shows that the datasets were assessed in accordance with the inclusion and exclusion criteria. The accessions were then obtained using the SRA Run Selector, and the sequence data files were downloaded using the SRA Toolkit. Using Bowtie2, the FASTA data were aligned to the reference genome (GRCm38) [30]. HTSeq was used to compute read counts for each gene in order to determine expression levels [31].

All the following computational analyses were carried out in the R environment. With the exception of the GSE83350 dataset, which contains a single animal per research group, read counts for each study were normalized, saved, and then pooled with the normalized counts of all other studies to generate a single DESeqDataSet. The design was also adjusted for the batch effect (in the study from which the data were obtained). Differential expression analysis was performed with the DESeq2 (1.30.1) [32] to compare all the infarcted groups with the sham. Genes that were differentially expressed were identified using a Log2FoldChange ≥ 2 criterion and a *p*-adjusted value < 0.05.

To perform functional enrichment analysis, the Cluster Profiler package [33] utilizing GO annotations with the MF category and adjusting the *p*-value with the Benjamin–Hochberg method was employed. We selected the MFs with involvement of the collagens of interest.

The ggplot2 (3.3.5) was employed to create the graphical representations [34] and Venn diagrams using the eulerr library in R Studio version 2022.12.0+353 (Boston, MA, USA).

### 4.2. Mouse MI Model

The Institutional Review Board authorized the animal protocols, which were carried out in accordance with European Parliament Directive 2010/63/EU (protocol number: GVRTE/2022/2209180).

Charles River Laboratories (Chatil-lon-sur-Chalaronne, France) provided the C57BL/6J mice, which were kept in pathogen-free settings with a constant temperature of 22 ± 2 °C and humidity of 60–65%, a 12 h light/dark cycle, and full access to regular chow and autoclaved water. Animals were 17 ± 2 weeks old and 1:1 female/male.

Briefly, before any surgical procedure, intraperitoneal buprenorphine (0.1 mg/kg) and meloxicam (0.3 mg/kg) were administered, and afterwards, mice were anesthetized by inhalation of 5% isoflurane (Abbott Laboratories, Chicago, IL, USA) delivered in 100% oxygen medical-grade air in an anesthetic chamber. Mice were then placed on the surgical board and subjected to tracheal intubation while connected to a rodent ventilator (Minivent type 845, Panlab Harvard Apparatus, Barcelona, Spain) set at a tidal volume of 200 μL, and a rate of 110 breaths per minute, supplemented with 100% oxygen and isoflurane (2%) at a flow rate of 0.2 L/min. Mice were maintained at a constant temperature of 37 °C with a heating pad. During the experiments, the electrocardiogram and animal temperature via a rectal probe were continuously monitored (Mouse Monitor S, Indus Instruments, Webster, TX, USA). After opening the thorax, a left minithoracotomy was performed at the height of the fourth intercostal space. Then, part of the pericardium was removed, and the coronary artery was located with a microscope for surgery. The occlusion process was carried out using a needle holder for microsurgery and 6-0 monofilament suture thread. The occlusion point was passed approximately 1–2 mm from the apex of the left atrium when in its normal position [35].

One control group and two independent MI experimental groups were formed: (1) non-reperfused MI (n = 8, permanent coronary ligation, without reperfusion) and (2) reperfused MI model (n = 8, transient 45 min occlusion of the coronary artery followed by reperfusion). In the non-reperfused MI model, the knot was tightened with the consequent occlusion of the coronary artery. In the reperfused MI model, a 23G tube was placed between the coronary artery and the 6-0 silk suture for later removal to allow complete reperfusion following the ischemic period. The tube was positioned through the sixth intercostal space to the exterior of the animal. In both MI models, once ischemia onset was confirmed both visually and on the electrocardiogram, the thorax was closed. In the reperfused MI model, the tube was removed after 45 min of occlusion to allow coronary reperfusion, which was confirmed by the resolution of ST-segment elevation on the electrocardiogram [35]. The control group (n = 6) was subjected to the same experimental protocol used in the MI groups, but without ligating the coronary artery.

Intraperitoneal buprenorphine (0.05 mg/kg, twice daily) and meloxicam (0.3 mg/kg, once daily) were administered for 5 days after surgery.

### 4.3. Human Sample Selection

This study conformed to the principles for the use of human subjects outlined in the Declaration of Helsinki. The study protocol was approved by the local Research Ethics Committee (Exp. 2022/315). The committee approved the exemption from informed consent.

Myocardial samples of eight patients with a reperfused infarction at chronic phase (more than 6 months after MI) were obtained from autopsies. A group of four patients with no evidence of cardiac fibrosis in the myocardium were also selected. Clinical and autopsy characteristics of the study patients are shown in Table 5 and Table 6.

### 4.4. Microscopic and Immunohistochemical Analysis

Human and murine myocardium tissue was fixed in 4% paraformaldehyde acid, embedded in paraffin, sectioned, and mounted on double gelatin-coated glass slides. Hematoxylin–eosin and Masson’s Trichrome staining was performed to corroborate the presence of infarcted areas [35,36].

For immune staining in human specimens, sections were first subjected to different methods of antigen retrieval (Table 7). For heat-induced epitope retrieval, sections were incubated with Tris/EDTA buffer (pH 9.0) (Dako, Glostrup, Denmark) at 121 °C for 3 min. Regarding proteolytic-induced epitope retrieval, samples were incubated with proteinase K (1 μg/mL, Sigma Aldrich, St. Louis, MO, USA) for 10 min at 37 °C. Subsequently, after unmasking and peroxidase blocking (0.3% H_2_O_2_), sections were incubated overnight (4 °C) with the specific primary antibody diluted in PBS/0.1% BSA. For immunohistochemistry, specific labeling was detected with a biotin-conjugated goat anti-mouse or goat anti-rabbit secondary antibody (1:500 dilution, Dako, Glostrup, Denmark) [35,36]. Further information about the primary antibodies, antigen retrieval method, and antibody concentration is detailed in Table 7.

### 4.5. Morphometric Quantification in the Infarcted Myocardium

A total of 40 samples were scanned using the Pannoramic 250 Flash III Scanner (3DHISTECH, Budapest, Hungary). For each sample and stain, five photographs at 63× magnification were taken using SlideViewer 2.6 Software (3DHISTECH, Budapest, Hungary) in independent fields of the infarct area. Images were morphometrically analyzed using Image ProPlus 7.0 software (Media Cybernetics Inc., Rockville, MD, USA) by a technician who was unaware of the study groups. Collagen presence was quantified as the percentage of area occupied by the selected object and the number of positive fibers [36,37]. The range used for segmentation is indicated in Table 8.

In mice samples, infarct size was calculated as the percentage of left ventricle occupied by fibrotic scars using samples stained with Masson’s Trichrome staining.

### 4.6. Quantitative Real-Time Polymerase Chain Reaction

To extract RNA, the RNeasy Plus Mini Kit (QIAGEN GmbH, Hilden, Germany) was employed following the manufacturer’s instructions. After reverse transcriptase reaction, gene expression was determined by real-time polymerase chain reaction using a 7900HT Fast Real-Time Polymerase Chain Reaction System (Applied Biosystems, ThermoFisher Scientific, Waltham, MA, USA). Threshold cycle (Ct) values were calculated in triplicate and normalized to the housekeeping gene 18S [37].

Specific pre-designed primers were used for the analysis of human and mouse samples (Applied Biosystems, ThermoFisher Scientific, Waltham, MA, USA). Further information about the primers employed in this study is detailed in Table 9.

### 4.7. Statistical Analysis

The Kolmogorov–Smirnov normality test was performed for each variable. Variables were expressed as mean ± SD. Unpaired Students’ *t*-test was used for comparisons, and correlations between infarct size and the mRNA expression of each collagen were calculated using a Spearman coefficient. Statistical significance was considered for a two-tailed *p*-value less than 0.05. SPSS 27.0 (SPSS, Inc., Chicago, IL, USA) was used throughout.

## 5. Conclusions

Our results demonstrate the presence of previously unreported collagen subunits, specifically types II, VIII, XI, and XII, in the infarcted myocardium using three different scenarios: (i) RNA-sequencing datasets from mice submitted to different ischemic times; (ii) experimental controlled mouse models of reperfused and non-reperfused MI; and (iii) myocardial samples of patients with chronic MI. These data could lay the groundwork for an enhanced understanding of the post-MI fibrotic scar composition, which will ultimately help unravel left ventricular remodeling following infarction.

## Figures and Tables

**Table 1:** Summary of GEO datasets for meta-analysis.

GEO DataSet	Reference	Number of Samples						
		Control	6 h Ischemia	1 Day Ischemia	3 Days Ischemia	7 Days Ischemia	14 Days Ischemia	21 Days Ischemia
GSE153494	[10]	3	3	3	3			
GSE153493	N/A	3		3			3	
GSE151834	[11]				4	4	4	8
GSE114695	[12]	9		3		3		3
GSE153485	[13]	10	5	5				
GSE154072	N/A						3	
GSE104187	[14]	4		2	2			
GSE83350	[15]	1			1			
Total		30	8	16	10	7	10	11

GEO: Gene Expression Omnibus. N/A: not available.

**Table 2:** Counts per million of genes encoding type I and II collagens and modulators of myocardial fibrosis at different times after coronary occlusion.

	Counts per Million						
Genes	Control	6 h Ischemia	1 Day Ischemia	3 Days Ischemia	7 Days Ischemia	14 Days Ischemia	21 Days Ischemia
*col1a1*	44.9 ± 32.0	20.0 ± 3.6	61.5 ± 32.8	2227.2 ± 1068.5 **	7995.1 ± 3871.1 **	1065.4 ± 1017.4 *	1167.8 ± 710.5 **
*col3a1*	144.0 ± 88.2	46.7 ± 16.4	181.0 ± 96.1	3696.8 ± 1365.6 **	13,659.5 ± 673.2 **	3965.3 ± 4010.0 *	42,433.6 ± 1973.6 **
*tgfb1*	12.4 ± 9.9	22.5 ± 18.6	44.2 ± 44.6 *	96.9 ± 61.6 **	59.6 ± 34.5 *	28.5 ± 21.8	29.3 ± 12.2 **
*ccn2*	91.7 ± 86.0	647.6 ± 407.8 *	562.6 ± 310.8 **	581.5 ± 233.7 **	2243.5 ± 614.7 **	957.9 ± 975.1 *	1651.0 ± 405.7 **
*acta2*	63.2 ± 20.2	53.1 ± 13.6	86.6 ± 65.0	330.2 ± 112.2 **	718.8 ± 247.9 **	142.7 ± 137.9	360.5 ± 66.0 **

* *p*-value < 0.05 ** *p*-value < 0.01 vs. control.

**Table 3:** mRNA expression of genes encoding type I and III collagens and modulators of myocardial fibrosis in non-reperfused and reperfused MI models in comparison to control animals.

Genes	Control	Non-Reperfused MI	Reperfused MI
*col1a1*	1.10 ± 0.53	27.75 ± 17.70 *	77.62 ± 52.38 *
*col3a1*	1.18 ± 0.70	24.83 ± 16.25 *	70.88 ± 43.19 *
*tgfb1*	1.04 ± 0.32	4.29 ± 2.12 **	5.43 ± 2.18 **
*ccn2*	1.34 ± 1.18	6.04 ± 4.27	14.43 ± 14.15
*acta2*	1.04 ± 0.34	2.79 ± 0.77 *	3.41 ± 1.12 **

* *p*-value < 0.05 ** *p*-value < 0.01 vs. control. MI: myocardial infarction.

**Table 4:** Correlation coefficients between infarct size and the transcriptomic levels of genes encoding type I and II collagens and modulators of myocardial fibrosis.

Genes	Spearman Rank-Order Correlation	*p*-Value
*col1a1*	0.89	0.00011
*col3a1*	0.89	0.00011
*tgfb1*	0.90	7.7 × 10^−5^
*ccn2*	0.91	3.7 × 10^−5^
*acta2*	0.90	5.4 × 10^−5^

**Table 5:** Clinical data and autopsy results of myocardial infarction patients.

Patient	Clinical Data	Autopsy Results
#1	78-year-old male Time elapsed since infarction: 5 years Cause of death: pneumonia	Infarct scar area: multiple foci in the lower wall of the left ventricle
#2	72-year-old female Time elapsed since infarction: 17 years Cause of death: aortic valve prosthesis dysfunction	Infarct scar area: 2.5 cm left ventricle
#3	62-year-old male Time elapsed since infarction: 1 year Cause of death: cerebral edema (diffuse large B-cell lymphoma)	Infarct scar area: 0.5 cm interventricular septum
#4	69-year-old male Time elapsed since infarction: 3 years Cause of death: multilobar pneumonia	Infarct scar area: left ventricle
#5	76-year-old male Time elapsed since infarction: 10 years Cause of death: myocardial infarction	Infarct scar area: ill-defined area in interventricular septum and posterior wall
#6	72-year-old male Time elapsed since infarction: 10 years Cause of death: refractory bradycardia and arrest after intervention	Infarct scar area: multiple foci in left ventricle
#7	84-year-old female Time elapsed since infarction: 1 year Cause of death: cardiogenic shock	Infarct scar area: 5 cm in the anterior wall of the left ventricle and interventricular septum
#8	76-year-old female Time elapsed since infarction: 1 year Cause of death: cardiac tamponade after bypass	Infarct scar area: multiple foci in the anterior side of the left ventricle

In patients 4, 5, 6, and 8, infarct size was not quantified but was visually described in the autopsy report.

**Table 6:** Clinical data and autopsy results of control subjects.

Patient	Clinical Data
#1	89-year-old male Cause of death: gastric adenocarcinoma
#2	62-year-old female Cause of death: hemorrhagic peritonitis
#3	93-year-old female Cause of death: pulmonary embolism
#4	66-year-old female Cause of death: acute pancreatitis with necrosis

**Table 7:** Experimental conditions for immunohistochemistry analysis depending on the primary antibody.

Marker	Specie	Concentration	Antigen Retrieval	Reference
Col2a1	Human	1:100	Enzymatic	Abcam St. Louis, MO, USA (ab185430)
Col8a1	Human	1:50	pH High	LSBio (Shirley, MA, USA) (aa583-743)
Col8a2	Human	1:100	pH High	Thermofisher (Waltham, MA, USA) (PA5-51280)
Col11a1	Human	1:100	pH High	Thermofisher (Waltham, MA, USA) (PA5-68410)
Col12a1	Human	1:50	pH High	Thermofisher (Waltham, MA, USA) (PA5-52655)

**Table 8:** Range used for segmentation of the five fibers in morphometric analysis.

Molecule	Area (μm^2^)	Diameter (μm)	Major Axis (μm)	Minor Axis (μm)
Col2a1	40–180	1–7	19–40	1.0–8
Col8a1	15–50	2–8	10–21	1.5–5
Col8a2	15–50	2–8	10–21	1.5–5
Col11a1	15–50	2–8	10–21	1.5–5
Col12a1	15–50	2–8	10–21	1.5–5

**Table 9:** References of the primers used in this study.

Gene	Specie	Reference
*col2a1*	Human	Hs01060337_g1
*col8a1*	Human	Hs05426099_s1
*col8a2*	Human	Hs07287101_m1
*col11a1*	Human	Hs01097671_g1
*col12a1*	Human	Hs01054146_m1
*col2a1*	Mouse	Mm01309565_m1
*col8a1*	Mouse	Mm01344185_m1
*col8a2*	Mouse	Mm02344867_g1
*col11a1*	Mouse	Mm00483387_m1
*col12a1*	Mouse	Mm01148576_m1
*col1a1*	Mouse	Mm00801666_g1
*col3a1*	Mouse	Mm00802300_m1
*acta2*	Mouse	Mm01546133_m1
*ccn2*	Mouse	Mm01192933_g1
*tgfb1*	Mouse	Mm00441727_g1

## Data Availability

Data is contained within the article.
